# Erector spinae plane block for low back pain in failed back surgery syndrome: a case report

**DOI:** 10.1186/s40981-018-0198-6

**Published:** 2018-08-27

**Authors:** Hidemasa Takahashi, Takeo Suzuki

**Affiliations:** Department of Anesthesiology, Tokyo Metropolitan Bokutoh Hospital, 4-23-15, Koto-bashi, Sumida-ku, Tokyo, 130-8575 Japan

**Keywords:** Failed back surgery syndrome, Low back pain, Paraspinal muscles, Ultrasound, Nerve block

## Abstract

**Background:**

Patients with failed back surgery syndrome (FBSS) experience chronic back pain following spinal surgery, and effective treatment is difficult because of multiple contributing factors.

**Case presentation:**

Here we report a case involving a 72-year-old woman who experienced recurrent low back pain after undergoing two back surgeries. She was treated with erector spinae plane (ESP) block, which affected the dorsal rami of the spinal nerves from T12 to L5. Pain relief lasted for approximately 10 h after the initial block, and successful low back pain relief was achieved after a total of three trials.

**Conclusions:**

ESP block, which is an easy and safe procedure, can be used to treat FBSS-associated low back pain.

## Background

Failed back surgery syndrome (FBSS) is a clinical condition involving the occurrence of persistent or recurring low back pain, radicular pain, or a combination of both after spinal surgery [1]. Patients with FBSS often experience difficulty in achieving adequate pain relief with conservative management (e.g., physical therapy and medication) and experience greater pain and worse quality of life than do patients with other chronic pain conditions [2]. FBSS may be caused by multiple etiological factors; therefore, effective treatment remains challenging [3, 4]. Erector spinae plane (ESP) block is a novel ultrasound-guided block that affects either the dorsal or both the dorsal and ventral rami of the spinal nerves [5, 6], and it could potentially be used to treat cases of low back pain. Melvin reported that ESP block was effective for perioperative analgesia in lumbosacral spine surgery [7]. Here we describe the first case, to the best of our knowledge, where ESP block successfully provided relief from chronic low back pain in a patient with FBSS.

We obtained approval for the present report from the ethics committee of the Tokyo Metropolitan Bokutoh Hospital. Written informed consent was obtained for the publication of this report.

## Case presentation

**Fig. 1:**
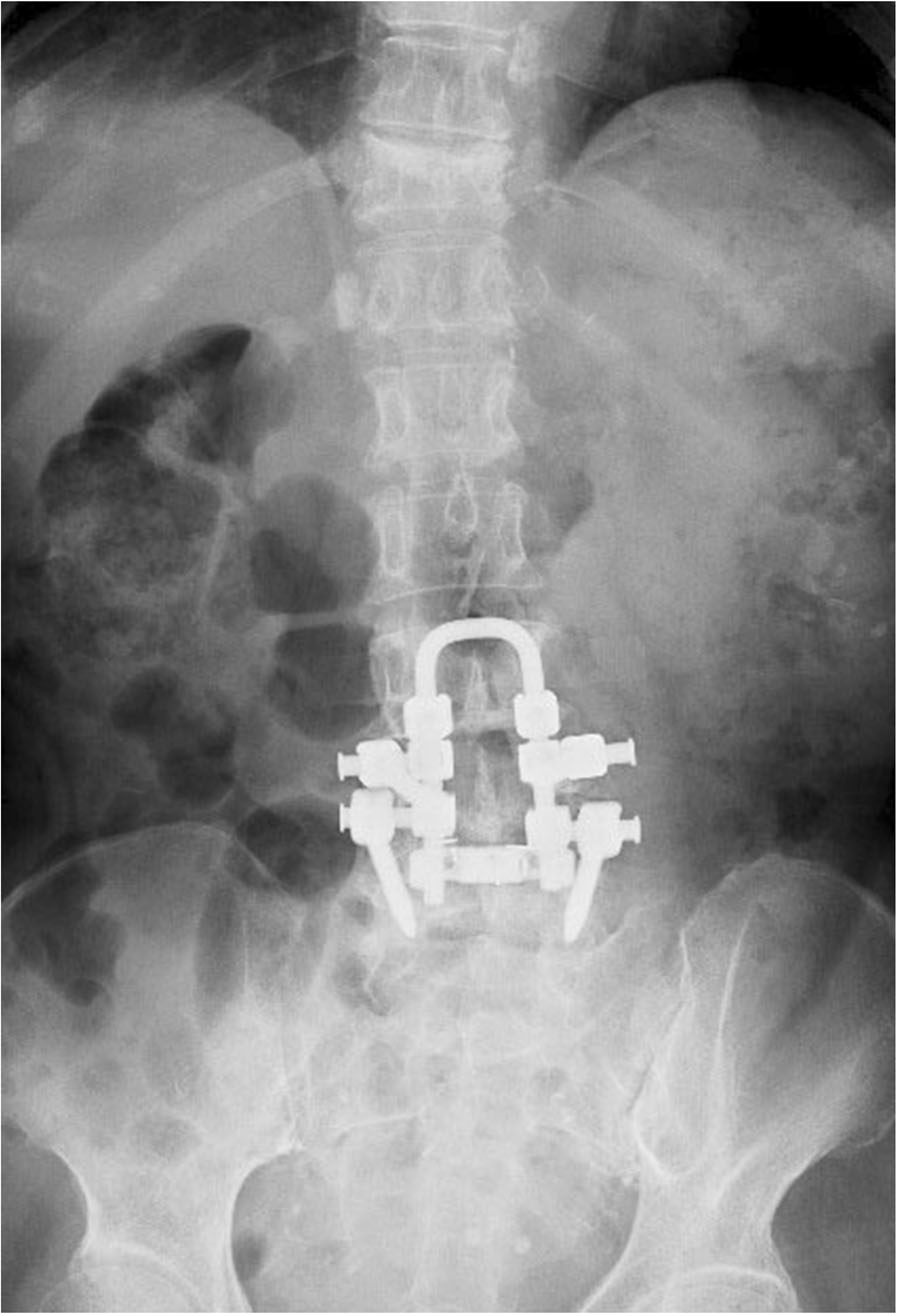
Radiograph showing lumbar spinal bone after two back surgeries in an elderly woman with failed back surgery syndrome

**Fig. 2:**
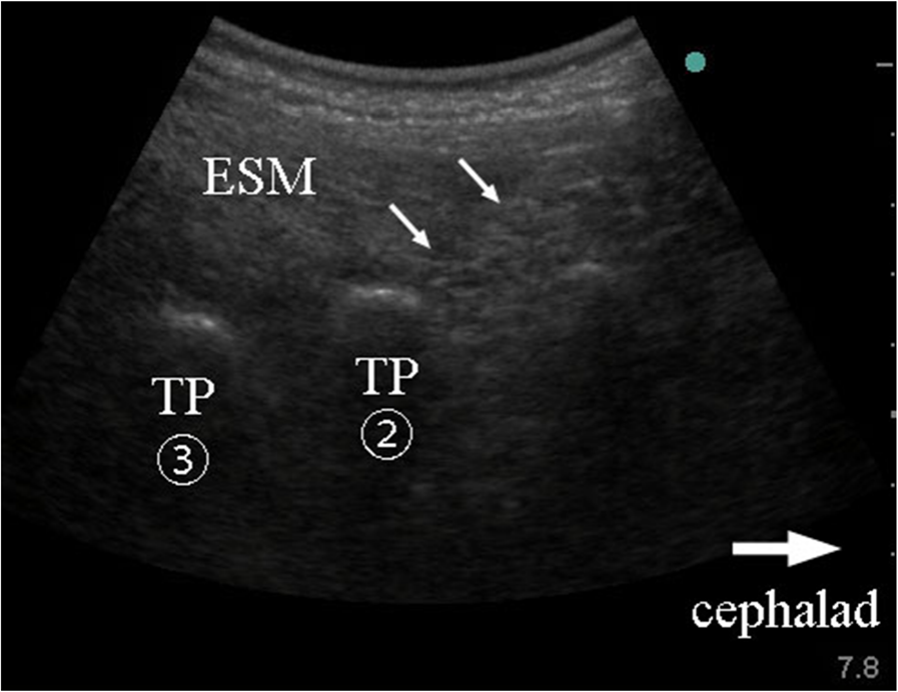
Ultrasound image for guidance during erector spinae plane (ESP) block for the treatment of low back pain in a patient with failed back surgery syndrome. TP(2), transverse process of L2; TP(3), transverse process of L3; ESM, erector spinae muscle white arrow, needle

A 72-year-old woman (40.3 kg, 139 cm) was referred to our pain clinic for the treatment of low back pain after two back surgeries. She had first undergone back surgery [posterior lumbar interbody fusion (PLIF at L4–5) plus spinal stabilization (L3)] for lumbar spinal canal stenosis 4 years prior to the current presentation. Two weeks after the initial surgery, she underwent reoperation because of screw placement errors, and her symptoms disappeared after surgery. However, her low back pain recurred after a fall 5 months before the current presentation. Paralysis of the lower limbs was not apparent after this episode. A sensory disturbance that had existed before the surgeries remained unchanged. No new lesions such as a lateral recess or foraminal stenosis, herniated nucleus pulposus, or fracture were found on radiographs or magnetic resonance images (Fig. 1). The previous medical institution prescribed acetaminophen and tramadol for low back pain and performed a caudal epidural block with 5 ml of 1% lidocaine and dexamethasone 1.65 mg. However, neither treatment provided pain relief, and the patient was referred to our pain clinic. She had several comorbidities, including diabetes mellitus, hypertension, renal dysfunction, hypothyroidism, rheumatic arthritis, and gastroesophageal regurgitation, and was prescribed 27 different drugs by clinicians from five different facilities. Accordingly, we decided not to use additional medication for first-line therapy because of polypharmacy concerns and renal dysfunction and performed bilateral ESP block with the patient in the prone position. A convex type transducer was placed in a longitudinal orientation at the level of the L2 transverse process, 3 cm lateral to the midline. The L4 and L5 transverse processes could not be identified because of the echogenic artifacts due to the surgical instruments. The posterior surface of the L2 transverse process was identified using an aseptic technique. After the puncture point was anesthetized with 2 ml of 1% lidocaine, the needle was inserted in the plane of the ultrasound beam in a cephalad to caudal direction. Following confirmation of the needle tip on the surface of the transverse process of L2, 20 ml of 0.1875% ropivacaine (fourfold dilution of commercial product) was injected into the target plane between the erector spinae muscles and the transverse process (Fig. 2). This procedure was repeated on the contralateral side. Twenty-five minutes later, the patient reported a warm feeling in her low back and almost complete relief from pain, which was approximately < 10% of its original severity. There was an area of diminished cold sensation extending from T12 to L5, with no change in the anterior and lateral abdomen. Unfortunately, the pinprick test was not performed. Pain relief lasted for approximately 10 h after the initial block. We repeated this procedure for a total of three times in a month. Finally, the patient reported that her daily baseline level of low back pain had diminished to < 40% of its original severity. She was satisfied with the extent of pain control and did not wish to undergo further treatments such as epiduroscopy or spinal cord stimulation (SCS).

## Discussion

To the best of our knowledge, this is the first case where successful low back pain relief was obtained with the use of ESP block in a patient with FBSS who experienced recurrent low back pain after undergoing two back surgeries.

ESP block is a novel, ultrasound-guided, peripheral nerve block that was first described for the treatment of chronic thoracic neuropathic pain [5]. The present report shows that ESP block at the lumbar vertebral level is as effective as that at the thoracic vertebral level, which is described in previous reports [5, 6]. ESP block is considered an extensive cutaneous sensory block because it affects either the dorsal rami or both the dorsal and ventral rami of the spinal nerves [5, 6]. In this case, a cold test determined that ESP block administered at the transverse process of L2 affected the dorsal rami of the lumbar spinal nerves from T12 to L5, but not the ventral rami. Although several studies have reported that ESP block affects both the dorsal and ventral rami of the spinal nerves [5, 8], a cadaver study showed that the ventral rami are not always blocked [9]. In addition, there may be differences between lumbar and thoracic ESP blocks [10]. Although FBSS is caused by multiple factors, low back pain in FBSS may be precipitated by the dorsal rami of the spinal cord [1]. Thus, ESP block is expected to alleviate low back pain in patients with FBSS. Indeed, as observed in the present case of FBSS, ESP block was highly effective in the treatment of low back pain. We used 20 ml of 0.1875% ropivacaine on the basis of previous studies [5, 6, 10]. Although there is no study on the appropriate dose of the local anesthetic, our dose was probably sufficient because adequate pain relief was obtained without adverse events. Several other blocks can affect the dorsal rami of the spinal nerves, such as the retroraminal block and thoracolumbar interfascial plane block [11, 12]. The injection site for both these blocks is close to that for ESP block. However, ESP block for FBSS, particularly that in the lumbar region, has not been adequately compared with these blocks. ESP block has several advantages. First, it is an easy and a safe procedure. The target is located on the transverse process, which gives a strong echo on ultrasound images and can be easily identified. Therefore, concerns regarding excessive advancement of the needle and inadvertent injection into the epidural or intrathecal space are limited. Second, the analgesic effect of ESP block is observed over a wider area. In this case, a cold test confirmed the effects between T12 and L5. This is an advantage over other regional analgesic techniques such as the trigger block and the medial branch block, which have effects limited to the injected area. Finally, surgical scar formation, which is a natural stage of tissue healing after surgery [3] and may limit drug spread to the target site, does not affect ESP block. The point at which drugs are injected for ESP block lies between the erector spinae muscle and the transverse process and is outside the surgical area, particularly that for the PLIF maneuver. Thus, there is likely to be minimal scar formation at the drug-injection site.

In general, the first choice for the treatment of FBSS is medication therapy [3]. However, this patient was already undergoing treatment with 27 different medications; therefore, she was not treated with additional medication because of polypharmacy concerns. Epidural block is a good choice for interventional therapy in pain clinics [3], but caudal epidural block was not effective in this case, probably because fibrotic adhesions formed by previous surgeries may have created separations within the epidural space [13], which interfered with the spread of the analgesic solution. As a more invasive treatment that can be used when conservative treatment is ineffective in cases of FBSS, SCS can play an important role in pain management [14]. A limitation of SCS is that some patients have reported complications, such as lead migration, local wound infection, pocket pain, loss of therapeutic effects, and cerebrospinal fluid leak with headache; these do not occur with ESP block [4]. Epiduroscopy is another option that may allow the physician to directly visualize the adhesions in the epidural space; this has also been reported to be effective [15]. However, this procedure is more invasive than ESP block and can only be performed at specialized institutions, particularly in Japan. For the above reasons, ESP block may be a valuable primary treatment option before the implementation of more invasive treatments for FBSS-associated low back pain.

There were three limitations to this case study. First, this report is just a case report, so it remains unclear whether ESP block is always an effective treatment for low back pain associated with FBSS, which can cause pain of various origins. For example, if low back pain originates inside the spinal canal, ESP block may not be effective. Further large-scale clinical studies are necessary for the generalization of our results. Second, this case involved a small woman, which makes it difficult to generalize our results. However, there is no relationship between low back pain in FBSS and physique. Thus, ESP could serve as a valuable treatment option for low back pain in patients with FBSS. Third, this report only showed the clinical effects of ESP block for FBSS, not the underlying mechanism. Further studies involving volunteers and cadavers subjected to lumbar surgery are necessary to clarify the mechanism.

In conclusion, the findings from this case suggest that ESP block is an easy and safe procedure and can serve as an effective treatment option for FBSS-associated low back pain.

## Abbreviations

ESP: Erector spinae plane
FBSS: Failed back surgery syndrome
PLIF: Posterior lumbar interbody fusion
SCS: Spinal cord stimulation
